# A meta-analysis of the association between adolescent pregnancy and the risk of gynecological cancers

**DOI:** 10.4178/epih.e2024094

**Published:** 2024-11-26

**Authors:** Bita Azmi-Naei, Fatemeh Shahbazi, Nazanin Azmi-Naei, Jalal Poorolajal

**Affiliations:** 1 Department of Epidemiology, School of Public Health, Hamadan University of Medical Sciences, Hamadan, Iran; 2 Department of Epidemiology, School of Public Health, Shahroud University of Medical Sciences, Shahroud, Iran; 3 Modeling of Noncommunicable Diseases Research Center, Hamadan University of Medical Sciences, Hamadan, Iran; 4 Research Center for Health Sciences, Hamadan University of Medical Sciences, Hamadan, Iran

**Keywords:** Pregnancy in adolescence, Gynecological neoplasms, Uterine cervical neoplasms, Ovarian neoplasms, Meta-analysis

## Abstract

**OBJECTIVES:**

Despite several investigations, the association between adolescent pregnancy and gynecological cancers has yet to be conclusively established. To further explore this association, we conducted a meta-analysis of observational studies.

**METHODS:**

We conducted a comprehensive search of databases such as PubMed, Web of Science, and Scopus to identify studies investigating the link between adolescent pregnancy and gynecologic cancers. This search continued until February 20, 2023. To assess the heterogeneity among the studies, we used the *I*^2^-statistics. We also explored the potential presence of publication bias using the Begg and Egger tests. The overall effect sizes were reported as either risk ratio or odds ratio, accompanied by a 95% confidence interval (CI), using a random-effects model.

**RESULTS:**

From an initial pool of 25,436 studies, a total of 76 studies involving 13,991,683 participants met the predefined eligibility criteria. The analysis indicated that the overall effect size for individuals having their first pregnancy at age 20 or older, compared to those having it before age 20, was 0.54 (95% CI, 0.50 to 0.59) for cervical cancer, 0.82 (95% CI, 0.77 to 0.88) for ovarian cancer, and 0.96 (95% CI, 0.89 to 1.04) for uterine cancer.

**CONCLUSIONS:**

Our findings suggest that experiencing one’s initial pregnancy at the age of 20 or above is associated with a significantly reduced risk of cervical and ovarian cancer. However, no significant association was found between first pregnancy at this age and uterine cancer.

## GRAPHICAL ABSTRACT

[Fig f6-epih-46-e2024094]

## Key Message

• First pregnancy at age 20 or older is associated with a reduced risk of cervical cancer.

• First pregnancy at age 20 or older is linked to a reduced risk of ovarian cancer.

• No significant association found between age of first pregnancy and uterine cancer.

## INTRODUCTION

Adolescent pregnancy remains a widespread issue worldwide [1]. It not only presents immediate challenges for young mothers and their children but also carries potential long-term health implications [2,3]. During pregnancy, adolescents experience unique physiological changes, such as rapid breast development and hormonal fluctuations. These changes may influence the risk of developing breast and gynecological cancers later in life [4,5]. Although research has explored the health outcomes of adolescent pregnancy, including preterm birth and low birth weight, less attention has been given to the long-term health consequences, particularly the risk of cancer [2,3]. In recent years, the potential link between adolescent pregnancy and the risk of developing certain gynecological cancers, such as cervical, ovarian, and endometrial cancers, has been the focus of attention. These cancers significantly impact women’s health worldwide, contributing to a considerable portion of cancer-related morbidity and mortality among women [6,7].

Investigating the potential link between adolescent pregnancy and the risk of gynecological cancers is essential for understanding the long-term health impacts on women. Although there is existing research on the relationship between adolescent pregnancy and gynecological cancers [8,9], our understanding of this connection is still limited and inconclusive. It is crucial from a public health perspective to explore this potential association further. Previous studies have examined this relationship [10–15], but the evidence remains inconclusive, largely due to limitations such as variations in study design, sample size, and methodological approaches.

A comprehensive meta-analysis is essential to synthesize the available data and provide a more robust estimate of the overall effect size. By examining the pooled evidence from multiple studies, we can address the knowledge gap and gain a clearer understanding of the long-term health consequences of adolescent pregnancy. This study aimed to synthesize the available evidence from observational studies to provide a comprehensive understanding of the potential long-term health consequences of adolescent pregnancies. These insights will be valuable for understanding the health implications of early pregnancy and can inform public health interventions and improve healthcare practices for young mothers.

## MATERIALS AND METHODS

### Eligibility criteria (PICOS)

(1) Population (P): The study population included women of reproductive age, with no restrictions based on race, ethnicity, or nationality. (2) Intervention/Exposure (I): The exposure of interest was adolescent pregnancy, which was defined as pregnancies occurring in girls between the ages of 10 and 19 [3]. (3) Control (C): The control group comprised women who had a previous pregnancy at the age of 20 or above. (4) Outcome (O): The outcome of interest was the occurrence of gynecological cancers, such as ovarian, endometrial, cervical, fallopian tube, vulvar, and vaginal cancers, which were confirmed through pathological examination. (5) Studies (S): We included observational studies, such as prospective cohort and case-control studies, that explored the relationship between gynecological cancers and adolescent pregnancy. There were no restrictions on publication status or language. To qualify for inclusion, studies needed to report the effect size using metrics like risk ratio (RR) or odds ratio (OR), along with their corresponding 95% confidence intervals (CIs). Studies were also considered eligible if they provided enough data to calculate these measures. Whenever possible, we used fully adjusted forms of the RR or OR that accounted for potential confounding factors such as age, race, smoking, alcohol consumption, physical activity, body mass index, education, socioeconomic status, use of oral contraceptives, hormone replacement therapy, breastfeeding, number of sexual partners, number of live births, diabetes, and history of stillbirths.

### Information sources and search strategy

We conducted a systematic search of the PubMed, Web of Science, and Scopus databases up to February 20, 2023. Additionally, we reviewed the reference lists of the included studies to identify any further eligible studies. Our search strategy employed a combination of keywords, searched as both “Text Word” and “MeSH terms” to ensure comprehensive coverage. The keywords included combinations of the following terms: (young or youth or teenage or teen or teenager or adolescent or adolescence or early) and (pregnancy or pregnancies or pregnant or parity) and (gynecologic or ovary or ovarian or uterine or uterus or womb or endometrium or endometrial or cervix or cervical or vagina or vaginal or vulva or vulvar or fallopian tubes) and (cancer or neoplasm or malignancy or tumor or carcinoma).

### Selection process

The search results from all databases were consolidated using EndNote software, and duplicate records were removed. Two authors (NAN and BAN), independently reviewed the titles and abstracts of the retrieved articles to exclude those that did not meet the inclusion criteria. The agreement between the 2 authors was substantial, with a kappa value of 78%. The full-texts of the potentially relevant studies were then obtained and subjected to a detailed evaluation to determine their eligibility for inclusion in the study.

### Data collection process

The data for the relevant studies were collected by 2 authors (FS and BAN), and were recorded in an electronic datasheet using Stata software. The information extracted included the first author’s name, publication year, country of study, language, mean or range of age, type of gynecological cancer (ovarian, endometrial, cervical, fallopian tube, vulvar, or vaginal), exposure (adolescent or adult pregnancy), study design (prospective cohort or case-control), sample size, analysis of potential confounders (adjusted or unadjusted), and effect size (RR, OR) along with their corresponding 95% CIs.

#### Assessment of risk of bias

The Newcastle-Ottawa Scale [16] was used to assess the methodological quality of the studies included in the analysis. This scale awards up to 9 stars to each study based on its quality across 3 key areas: the selection of study groups, the comparability of these groups, and the determination of either the exposure or outcome of interest. Studies that achieved a score of 7 or more stars were considered high-quality, whereas those scoring fewer than 7 stars were deemed low-quality.

#### Effect measures and synthesis methods

In this systematic review, the primary measure of interest was the RR for prospective cohort studies and the OR for case-control studies. These effect measures were pooled together separately, and the overall summary measure was reported as either the RR or OR, employing a random-effects model [17]. The data analysis was performed at a significance level of 0.05 using Stata version 14.2 (StataCorp., College Station, TX, USA).

#### Assessment of heterogeneity and publication bias

To explore possible heterogeneity among the studies analyzed, both the chi-square test [18] and the *I*^2^-statistic [19] were used. The *I*^2^-statistic was employed to examine the degree of heterogeneity, where values below 50% indicated low heterogeneity, values ranging from 50% to 74% indicated moderate heterogeneity and values of 75% or higher indicated high heterogeneity [20]. To evaluate the possible existence of publication bias, both the Egger test [21] and the Begg test [22] were used.

#### Sensitivity analysis

When moderate to high between-study heterogeneity was detected (*I*^2^≥50%), we performed a sensitivity analysis using the MetaPlot Stata tool [23,24]. The MetaPlot tool is based on a sequential algorithm [25] and offers a graphical method to identify specific studies that contribute significantly to the overall heterogeneity. The sequential algorithm utilized in MetaPlot involves conducting “leave-one-out” sensitivity analyses iteratively, excluding 1 study at a time, to assess the influence of individual studies on the overall results of the meta-analysis. This approach provides a straightforward and efficient way to evaluate the impact of individual studies on the overall findings.

### Ethics statement

No human or animal subjects were involved in this study.

## RESULTS

### Study selection and characteristics

**Figure 1:**
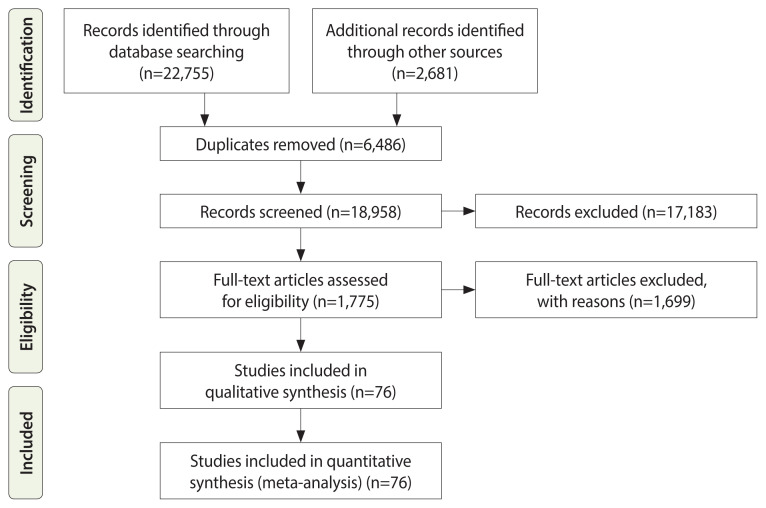
Flow of information through the various phases of the systematic review.

Initially, a total of 25,436 studies were identified, with 22,755 derived from electronic database searches and 2,681 found through screening the reference lists of included studies. After removing duplicates and excluding ineligible studies, 76 studies involving 13,991,683 participants were selected for the meta-analysis (Figure 1, Supplementary Material 1). These studies included 35 on cervical cancer, 27 on ovarian cancer, and 21 on uterine cancer. It is important to note that some studies covered multiple cancer types, which is why the number of individual studies exceeds the total number of included studies. Notably, no study was found that explored the association between adolescent pregnancy and the risk of cancer in the fallopian tube, vulva, or vagina.

### Synthesis of results

**Figure 2:**
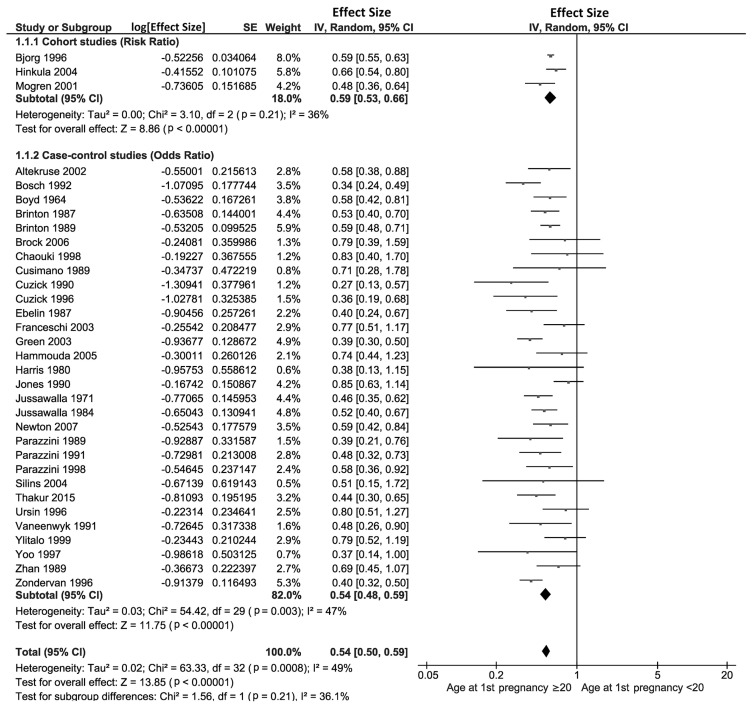
Forest plot of prospective cohort and case-control studies assessing adolescent pregnancy and cervical cancer risk. SE, standard error; CI, confidence interval; df, degrees of freedom.

Figure 2 illustrates the relationship between adolescent pregnancy and the risk of cervical cancer. Data from 3 prospective cohort studies showed that the RR for women who had their first pregnancy at age 20 or older, compared to those under 20, was 0.59 (95% CI, 0.53 to 0.66). This indicates that women who delayed their first pregnancy until they were 20 or older had a 41% reduced risk of developing cervical cancer (p<0.001). The heterogeneity among these cohort studies was low, as reflected by an *I*^2^-value of 36%. Additionally, an analysis of 30 case-control studies revealed that the OR for having the first pregnancy at age 20 or older versus younger than 20 was 0.54 (95% CI, 0.48 to 0.59). This suggests that women who delayed their first pregnancy until after 20 years of age had a 46% lower chance of developing cervical cancer (p<0.001). The heterogeneity among the case-control studies was also low, indicated by an *I*^2^-value of 47%.

**Figure 3:**
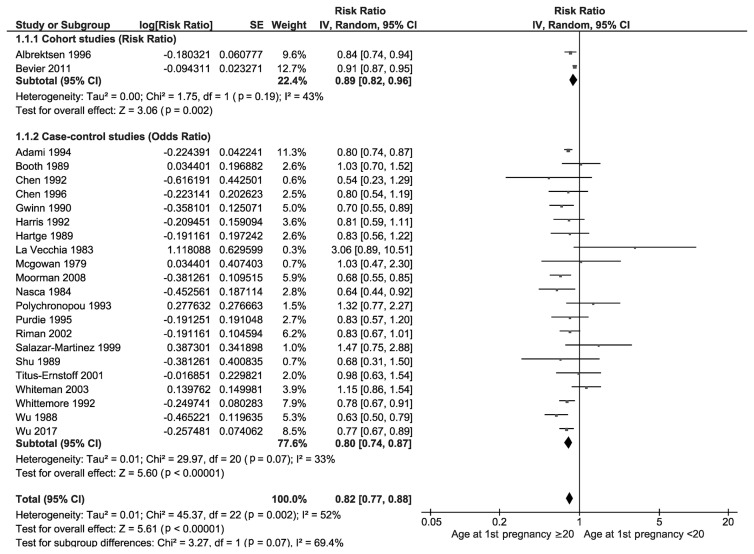
Forest plot of prospective cohort and case-control studies assessing adolescent pregnancy and ovarian cancer risk. SE, standard error; CI, confidence interval; df, degrees of freedom.

Figure 3 displays the association between adolescent pregnancy and the risk of ovarian cancer. Data from 2 prospective cohort studies showed that the RR for women who had their first pregnancy at age 20 or older, compared to those who had it before age 20, was 0.89 (95% CI, 0.82 to 0.96). This indicates that women who delayed their first pregnancy until they were 20 or older have an approximately 11% reduced risk of developing ovarian cancer (p=0.002). The heterogeneity among these cohort studies was low, with an *I*^2^-value of 43%. Furthermore, an analysis of 21 case-control studies revealed that the overall OR for having a first pregnancy at age 20 or older, compared to younger than 20, was 0.80 (95% CI, 0.74 to 0.87). This suggests that women who delayed their first pregnancy until age 20 or older had about a 20% lower chance of developing ovarian cancer (p<0.001). The heterogeneity among the case-control studies was also low, with an *I*^2^-value of 33%.

**Figure 4:**
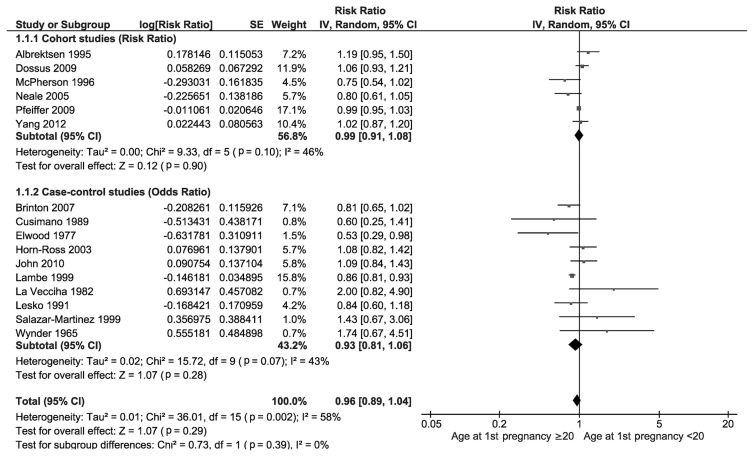
Forest plot of prospective cohort and case-control studies assessing adolescent pregnancy and uterine cancer risk. SE, standard error; CI, confidence interval; df, degrees of freedom.

Figure 4 presents the relationship between adolescent pregnancy and the risk of uterine cancer. An analysis of 6 prospective cohort studies revealed that the overall RR for women who had their first pregnancy at age 20 or older, compared to those under 20, was 0.99 (95% CI, 0.91 to 1.08). This suggests that women who had their first pregnancy at 20 or older had an approximately 1% lower probability of developing uterine cancer, although this result was not statistically significant (p=0.90). The heterogeneity among these cohort studies was low, with an *I*^2^-value of 46%. Additionally, an analysis of 10 case-control studies showed that the overall OR for having a first pregnancy at age ≥20 years compared to <20 years was 0.93 (95% CI, 0.81 to 1.06). This indicates that women who had their first pregnancy at age 20 or older had an approximately 7% lower probability of developing uterine cancer, though these findings were also not statistically significant (p=0.28). The heterogeneity among the case-control studies was similarly low, with an *I*^2^-value of 43%.

### Sensitivity analysis

To address the observed variability among the included studies, we conducted a sensitivity analysis using a sequential technique. This process systematically excluded individual studies to assess their impact on overall homogeneity. Through this iterative approach, we achieved homogeneity, as indicated by an *I*^2^-value below 50%. Table 1 shows that the association between adolescent pregnancy and certain gynecological cancers (though not all) exhibited a slight decrease in strength after the sensitivity analysis, compared to the initial results. This analysis offered valuable insights into the influence of study heterogeneity on the overall effect sizes.

### Publication bias

**Figure 5:**
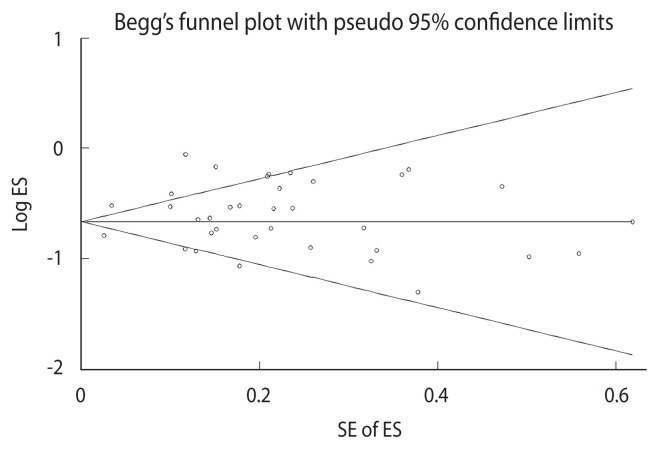
Funnel plot of prospective cohort and case-control studies assessing adolescent pregnancy and cervical cancer risk. ES, effect size; SE, standard error.

Figure 5 demonstrates the lack of significant publication bias in the association between adolescent pregnancy and cervical cancer, as evidenced by the results of the Begg (p=0.402) and Egger (p=0.201) tests.

The association between adolescent pregnancy and ovarian cancer showed no statistical significance in the results of the Begg (p=0.084) and Egger (p=0.628) tests, indicating a lack of significant publication bias.

The association between adolescent pregnancy and uterine cancer was analyzed using the Begg (p=1.000) and Egger (p=0.304) tests. The results were not statistically significant, indicating no evidence of publication bias.

### Racial distribution

The analysis included a total of 76 studies, spread across 6 continents. Europe was the most represented with 32 studies (42.1%), followed by North America with 25 studies (32.9%). South America contributed 3 studies (3.9%), Asia 9 studies (11.8%), Africa 1 study (1.3%), and Australia 3 studies (3.9%). Additionally, 3 studies were conducted in intercontinental regions.

## DISCUSSION

The findings of this meta-analysis revealed that women who had their first pregnancy at age 20 or older experienced a significantly lower risk of cervical cancer (41% lower), an 11% reduced risk of ovarian cancer, and a slight, though not statistically significant, reduction in the risk of uterine cancer. These results underscore the potential protective effect of delaying pregnancy until age 20 or older with respect to these gynecological cancers.

The precise mechanisms underlying the relationship between adolescent pregnancy and an increased risk of certain gynecological cancers remain unclear. However, several hypotheses have been proposed to explain this correlation. Adolescent mothers may engage in risky behaviors, such as smoking, substance abuse, poor nutrition, and inadequate healthcare utilization, all of which can contribute to an overall higher risk of cancer [26,27]. Additionally, the cervix continues to mature throughout adolescence. Early age at first sexual intercourse and pregnancy before the cervix has fully matured may increase susceptibility to certain infections, such as human papillomavirus infection, a known risk factor for cervical cancer [8,28,29]. Furthermore, there are biological explanations for why becoming pregnant at a later age might lower the risk of endometrial cancer. One reason is that prolonged exposure to progesterone during pregnancy is particularly beneficial for older women. Moreover, older women who have been pregnant may experience fewer anovulatory cycles, which can also contribute to the protective effect against endometrial cancer [4,30].

While this meta-analysis established a positive association between adolescent pregnancy and certain gynecological cancers, the risk associated with these cancers is complex. Multiple risk and protective factors interact to influence cancer development, and these factors are interconnected rather than independent. Risk factors increase the likelihood of cancer, whereas protective factors reduce it. When these factors are balanced, or when protective factors outweigh the risks, the likelihood of developing cancer is reduced [31]. Therefore, the role of adolescent pregnancy in the incidence of gynecological cancers needs to be evaluated in conjunction with other influential factors.

This review has several limitations and potential biases that should be considered when interpreting the results. First, despite efforts to manage heterogeneity through sensitivity analysis, variations in study design, population characteristics, and other factors may still contribute to heterogeneity, limiting the comparability of the included studies. Second, although adjustments were made to control for confounding variables in the reported effect sizes, it is possible that not all potential confounders affecting the relationship between adolescent pregnancy and gynecological cancers were fully accounted for. Uncontrolled confounders could introduce bias and affect the validity of the results. Third, certain specific gynecological cancers, such as cancers of the fallopian tube, vulva, or vagina, were underrepresented in the studies included. Consequently, the findings may not be generalizable to these specific types of cancer. Fourth, while the assessment of publication bias did not reveal significant bias, the possibility remains that unpublished studies or studies with non-significant results were excluded, potentially skewing the overall findings. Fifth, the studies included in this meta-analysis varied in quality, and some may have been subject to biases or limitations that could affect the reliability of their findings. Lastly, this meta-analysis identifies associations between adolescent pregnancy and gynecological cancers but does not establish causality. The observed associations could be influenced by other unaccounted factors.

The majority of studies included in the meta-analysis were conducted between the 1980s and 1990s, which might raise questions about their current relevance. Nevertheless, the basic principles regarding early pregnancy and its link to the risk of gynecological cancer have remained unchanged. This consistency is due to the stable nature of the underlying biological mechanisms and risk factors involved. Despite the age of these studies, the public health implications of this relationship remain substantial. While newer studies might offer fresh perspectives, obtaining high-quality data in this area can often be challenging. Therefore, our meta-analysis provides a thorough review of the available evidence, establishing a solid base for understanding the connection between early pregnancy and the risk of gynecological cancer. Further research, involving larger sample sizes and more recent data, will improve our knowledge and refine this critical field of study.

This study primarily focused on the association between age at first pregnancy and the risk of gynecological cancer. However, it is important to acknowledge that the age at first sexual intercourse may also play a significant role. Although these 2 factors are often correlated, they are not always directly linked. Variables such as contraceptive use, fertility, and personal intentions can influence the timing of pregnancy, even within the context of an established sexual relationship. Future research that explores the independent effects of both age at first sexual intercourse and age at first pregnancy could provide a more comprehensive understanding of the risk factors for gynecological cancers. Additionally, investigating the potential interactions between these 2 variables could illuminate the complex interplay of factors that influence cancer development.

While the connection between adolescent pregnancy and gynecological cancers involves various influencing factors, the outcomes of this review may have significant implications for public health policy. Policymakers should prioritize prevention-focused interventions to reduce the incidence of adolescent pregnancies and subsequent gynecological cancers. These interventions could include comprehensive gender education programs in schools and community settings, promoting healthy relationships, and raising awareness about the importance of contraception and regular gynecological screenings. Public health policies should also aim to improve access to healthcare services for adolescents, particularly in underserved communities. Efforts could involve expanding healthcare coverage, ensuring the availability of affordable reproductive health services, and establishing adolescent-friendly clinics that cater to the unique needs and preferences of young individuals. Finally, conducting meticulously designed prospective cohort studies could yield more robust evidence regarding the association between adolescent pregnancy and the risk of gynecological cancer. This approach allows for improved control over confounding factors, thereby enhancing the reliability and depth of the findings.

## CONCLUSION

Our findings suggest that women who have their first pregnancy at age 20 or older are associated with a significantly reduced risk of cervical and ovarian cancer. However, no significant association was observed between the age at first pregnancy and the risk of uterine cancer. It is important to note that the studies included in this analysis were observational, which constrains our capacity to determine a definitive cause-and-effect relationship. Additionally, there was significant heterogeneity among the studies. Therefore, further research, especially prospective studies, is necessary to explore the potential link between adolescent pregnancy and gynecological cancers.

## Figures and Tables

**Figure f6-epih-46-e2024094:**
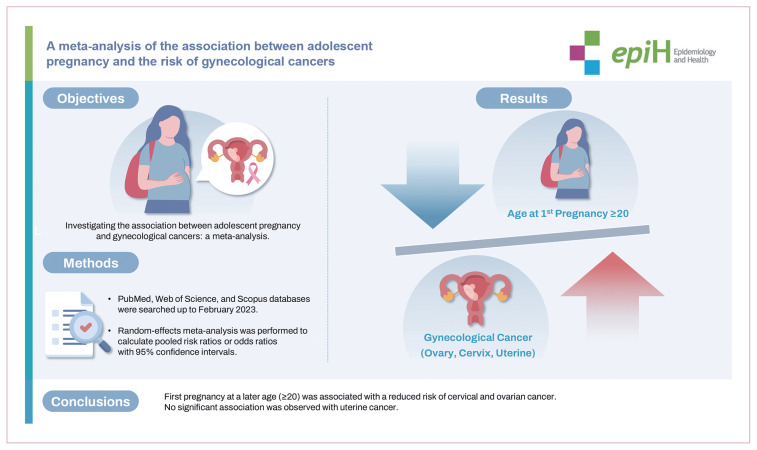


**Table 1:** Results of the sensitivity analysis for the association between adolescent pregnancy and risk of gynecological cancers

Analysis	Original analysis			Sensitivity analysis		
	n	*I*^2^ (%)	RR (95% CI)	n	*I*^2^ (%)	RR (95% CI)
Cohort studies						
Cervical cancer	4	94	0.54 (0.44, 0.66)	3	36	0.59 (0.53, 0.66)
Ovarian cancer	4	93	0.87 (0.70, 1.08)	2	43	0.89 (0.82, 0.96)
Uterine cancer	9	93	0.97 (0.84, 1.13)	6	46	0.99 (0.91, 1.08)
Case-control studies						
Cervical cancer	31	61	0.55 (0.49, 0.62)	30	47	0.54 (0.48, 0.59)
Ovarian cancer	23	71	0.89 (0.79, 0.99)	21	33	0.80 (0.74, 0.87)
Uterine cancer	12	74	0.84 (0.75, 0.94)	10	43	0.93 (0.81, 1.06)

RR, risk ratio; OR, odds ratio; CI, confidence interval.
