# Supplementary material for: A meta-analysis of the association between adolescent pregnancy and the risk of gynecological cancers
Source: Epidemiol Health. 2024 Nov 26;46:e2024094. doi: 10.4178/epih.e2024094 (PMC11840396; doi:10.4178/epih.e2024094)
Supplement: Supplementary file 1 [file epih-46-e2024094-Supplementary.docx]

**Supplementary Material 1**: Characteristics of included studies, sorted by first authors' names [1-76]

| **ID** | **1^st^ Autor year** | **Country** | **Age mean/range** | **Type of cancer** | **Study design** | **Adjustment** | **Sample size** | **NOS score** | **Quality** |
| --- | --- | --- | --- | --- | --- | --- | --- | --- | --- |
|  | Adami 1994 | Sweden | Missing | Ovary | Case-control | Adjusted | 20,916 | 8 | High |
|  | Albrektsen 1995 | Norway | 30-56 | Uterine | Cohort | Adjusted | 765,756 | 8 | High |
|  | Albrektsen 1996 | Norway | 20-56 | Ovary | Cohort | Adjusted | 1,145,076 | 8 | High |
|  | Altekruse 2002 | USA | 21-67 | Cervix | Case-control | Adjusted | 446 | 7 | High |
|  | Bevier 2011 | Sweden | Missing | Ovary, Uterine | Cohort | Adjusted | 5,759,120 | 7 | High |
|  | Bjorg 1996 | Norway | 20-56 | Cervix | Cohort | Unadjusted | 1,300,000 | 7 | High |
|  | Booth 1989 | UK | 51.74 | Ovary | Case-control | Adjusted | 686 | 6 | Low |
|  | Bosch 1992 | Spain & Colombia | 50.08 | Cervix | Case-control | Unadjusted | 823 | 6 | Low |
|  | Boyce 1977 | USA | 33.20 | Cervix | Case-control | Adjusted | 1,378 | 8 | High |
|  | Boyd 1964 | UK | 51.90 | Cervix | Case-control | Adjusted | 891 | 5 | Low |
|  | Brinton 1987 | USA | 44.82 | Cervix | Case-control | Adjusted | 1,122 | 5 | Low |
|  | Brinton 1989 | 4 nations | 46.50 | Cervix | Case-control | Adjusted | 2,189 | 8 | High |
|  | Brinton 2007 | Poland | 57.20 | Uterine | Case-control | Adjusted | 2,476 | 8 | High |
|  | Brock 2006 | Australia | 18-65 | Cervix | Case-control | Adjusted | 313 | 4 | Low |
|  | Chaouki 1998 | Morocco | 45.32 | Cervix | Case-control | Adjusted | 417 | 8 | High |
|  | Chen 1992 | China | 48.83 | Ovary | Case-control | Adjusted | 336 | 7 | High |
|  | Chen 1996 | USA | 50.58 | Ovary | Case-control | Adjusted | 748 | 6 | Low |
|  | Chiaffarino 2001 | Italy | 17-79 | Ovary | Case-control | Adjusted | 3,442 | 8 | High |
|  | Cusimano 1989 | Italy | 58.03 | Cervix, Uterine | Case-control | Unadjusted | 480 | 7 | High |
|  | Cuzick 1990 | UK | <40 | Cervix | Case-control | Unadjusted | 1,330 | 7 | High |
|  | Cuzick 1996 | UK | 33.55 | Cervix | Case-control | Adjusted | 362 | 8 | High |
|  | Dossus 2009 | 10 nations | 50.50 | Uterine | Cohort | Adjusted | 306,018 | 8 | High |
|  | Ebelin 1987 | Germany | 20-54 | Cervix | Case-control | Adjusted | 404 | 6 | Low |
|  | Elwood 1977 | USA | Missing | Uterine | Case-control | Unadjusted | 1,410 | 5 | Low |
|  | Franceschi 2003 | India | Missing | Cervix | Case-control | Adjusted | 418 | 7 | High |
|  | Green 2003 | Uk | 20-44 | Cervix | Case-control | Unadjusted | 1,494 | 8 | High |
|  | Greggi 2000 | Italy | 13-80 | Ovary | Case-control | Adjusted | 1,308 | 7 | High |
|  | Gwinn 1990 | USA | 20-54 | Ovary | Case-control | Adjusted | 4,269 | 6 | Low |
|  | Hammouda 2005 | 10 nations | 30-88 | Cervix | Case-control | Adjusted | 400 | 7 | High |
|  | Harris 1980 | UK | 35.60 | Cervix | Case-control | Unadjusted | 659 | 7 | High |
|  | Harris 1992 | USA | Missing | Ovary | Case-control | Adjusted | 4,471 | 8 | High |
|  | Hartge 1989 | USA | 54.56 | Ovary | Case-control | Adjusted | 639 | 8 | High |
|  | Hinkula 2004 | Finland | Missing | Cervix | Cohort | Adjusted | 86,978 | 8 | High |
|  | Horn-Ross 2003 | USA | 35-79 | Uterine | Case-control | Adjusted | 970 | 7 | High |
|  | John 2010 | USA | 35-79 | Uterine | Case-control | Adjusted | 915 | 6 | Low |
|  | Jones 1990 | USA | 20-74 | Cervix | Case-control | Adjusted | 1,094 | 7 | High |
|  | Jussawalla 1971 | India | Missing | Cervix | Case-control | Adjusted | 1,006 | 7 | High |
|  | Jussawalla 1984 | India | Missing | Cervix | Case-control | Adjusted | 2,328 | 8 | High |
|  | La Vecchia 1983 | Italy | <69 | Ovary | Case-control | Adjusted | 722 | 7 | High |
|  | La Vecciha 1982 | Italy | 33-84 | Uterine | Case-control | Adjusted | 520 | 5 | Low |
|  | Lambe 1999 | Sweden | Missing | Uterine | Case-control | Adjusted | 29,023 | 6 | Low |
|  | Lesko 1991 | USA | 30-69 | Uterine | Case-control | Adjusted | 1,176 | 7 | High |
|  | Mcgowan 1979 | USA | 49.89 | Ovary | Case-control | Unadjusted | 347 | 4 | Low |
|  | McPherson 1996 | USA | 61.80 | Uterine | Cohort | Adjusted | 24,848 | 6 | Low |
|  | Mogren 2001 | Sweden | Missing | Cervix, Ovary, Uterine | Cohort | Adjusted | 40,951 | 7 | High |
|  | Moorman 2008 | USA | 20-74 | Ovary | Case-control | Unadjusted | 1,863 | 6 | Low |
|  | Nasca 1984 | USA | 55.13 | Ovary | Case-control | Adjusted | 1,209 | 8 | High |
|  | Neale 2005 | Sweden | Missing | Cervix, Ovary, Uterine | Cohort | Adjusted | 1,234,967 | 8 | High |
|  | Newton 2007 | Uganda | >15 | Cervix | Case-control | Unadjusted | 702 | 6 | Low |
|  | Parazzini 1989 | Italy | 18-74 | Cervix | Case-control | Adjusted | 984 | 6 | Low |
|  | Parazzini 1991 | Italy | 18-59 | Cervix | Case-control | Adjusted | 689 | 5 | Low |
|  | Parazzini 1998 | Italy | 16-44 | Cervix | Case-control | Adjusted | 518 | 6 | Low |
|  | Pfeiffer 2009 | sweden | 20-72 | Uterine | Cohort | Adjusted | 2,674,465 | 7 | High |
|  | Pocobelli 2011 | USA | 59.76 | Uterine | Case-control | Adjusted | 3,846 | 6 | Low |
|  | Polychronopou 1993 | Greece | <75 | Ovary | Case-control | Adjusted | 389 | 6 | Low |
|  | Purdie 1995 | Australia | 55.14 | Ovary | Case-control | Unadjusted | 1,684 | 6 | Low |
|  | Riman 2002 | Sweden | 63.12 | Ovary | Case-control | Adjusted | 5,174 | 6 | Low |
|  | Salazar-Martinez 1999 | Mexico | 54.67 | Ovary, Uterine | Case-control | Adjusted | 837 | 6 | Low |
|  | Shu 1989 | China | 49.00 | Ovary | Case-control | Adjusted | 458 | 6 | Low |
|  | Silins 2004 | Latvia | 52.78 | Cervix | Case-control | Adjusted | 462 | 7 | High |
|  | Thakur 2015 | India | Missing | Cervix | Case-control | Adjusted | 452 | 6 | Low |
|  | Titus-Ernstoff 2001 | USA | 20-74 | Ovary | Case-control | Unadjusted | 1,086 | 5 | Low |
|  | Trabert 2019 | Uk | 57.80 | Uterine | Case-control | Adjusted | 134,673 | 7 | High |
|  | Ursin 1996 | USA | 44.96 | Cervix | Case-control | Adjusted | 581 | 5 | Low |
|  | Vaneenwyk 1991 | USA | >18 | Cervix | Case-control | Adjusted | 204 | 6 | Low |
|  | Wernli 2006 | China | >30 | Uterine | Cohort | Adjusted | 267,400 | 8 | High |
|  | Whiteman 2003 | Australia | 18-79 | Ovary | Case-control | Adjusted | 1,343 | 8 | High |
|  | Whittemore 1992 | USA | Missing | Ovary | Case-control | Unadjusted | 11,090 | 4 | Low |
|  | Wu 1988 | USA | 54.43 | Ovary | Case-control | Adjusted | 1,310 | 6 | Low |
|  | Wu 2017 | USA | Missing | Ovary | Case-control | Adjusted | 3,972 | 8 | High |
|  | Wynder 1965 | USA | 59.30 | Uterine | Case-control | Unadjusted | 312 | 6 | Low |
|  | Yang 2012 | USA | 61.60 | Uterine | Cohort | Unadjusted | 114,409 | 4 | Low |
|  | Ylitalo 1999 | Sweden | 25-49 | Cervix | Case-control | Adjusted | 844 | 8 | High |
|  | Yoo 1997 | Korea | 20-60 | Cervix | Case-control | Adjusted | 1,030 | 8 | High |
|  | Zhan 1989 | China | >25 | Cervix | Case-control | Adjusted | 654 | 6 | Low |
|  | Zondervan 1996 | UK | <45 | Cervix | Case-control | Adjusted | 3,401 | 8 | High |

**References**

1. Adami HO, Hsieh CC, Lambe M, Trichopoulos D, Leon D, Persson I, et al. Parity, age at first childbirth, and risk of ovarian cancer. Lancet. 1994; 344:1250-4.

2. Albrektsen G, Heuch I, Kvåle G. Reproductive factors and incidence of epithelial ovarian cancer: a Norwegian prospective study. Cancer Causes Control. 1996; 7:421-7.

3. Albrektsen G, Heuch I, Tretli S, Kvåle G. Is the risk of cancer of the corpus uteri reduced by a recent pregnancy? A prospective study of 765,756 Norwegian women. Int J Cancer. 1995; 61:485-90.

4. Altekruse SF, Lacey JV, Jr., Brinton LA, Gravitt PE, Silverberg SG, Barnes WA, Jr., et al. Comparison of human papillomavirus genotypes, sexual, and reproductive risk factors of cervical adenocarcinoma and squamous cell carcinoma: Northeastern United States. Am J Obstet Gynecol. 2003; 188:657-63.

5. Bevier M, Sundquist J, Hemminki K. Does the time interval between first and last birth influence the risk of endometrial and ovarian cancer? Eur J Cancer. 2011; 47:586-91.

6. Bjørge T, Kravdal O. Reproductive variables and risk of uterine cervical cancer in Norwegian registry data. Cancer Causes Control. 1996; 7:351-57.

7. Booth M, Beral V, Smith P. Risk factors for ovarian cancer: a case-control study. Br J Cancer. 1989; 60:592-8.

8. Bosch FX, Muñoz N, de Sanjosé S, Izarzugaza I, Gili M, Viladiu P, et al. Risk factors for cervical cancer in Colombia and Spain. Int J Cancer. 1992; 52:750-8.

9. Boyce JG, Lu T, Nelson JH, Jr., Fruchter RG. Oral contraceptives and cervical carcinoma. Am J Obstet Gynecol. 1977; 128:761-6.

10. Boyd JT, Doll R. A STUDY OF THE AETIOLOGY OF CARCINOMA OF THE CERVIX UTERI. Br J Cancer. 1964; 13:419-34.

11. Brinton LA, Hamman RF, Huggins GR, Lehman HF, Levine RS, Mallin K, et al. Sexual and reproductive risk factors for invasive squamous cell cervical cancer. J Natl Cancer Inst. 1987; 79:23-30.

12. Brinton LA, Reeves WC, Brenes MM, Herrero R, de Britton RC, Gaitan E, et al. Parity as a risk factor for cervical cancer. Am J Epidemiol. 1989; 130:486-96.

13. Brinton LA, Sakoda LC, Lissowska J, Sherman ME, Chatterjee N, Peplonska B, et al. Reproductive risk factors for endometrial cancer among Polish women. Br J Cancer. 2007; 96:1450-6.

14. Brock KE, Berry G, Brinton LA, Kerr C, MacLennan R, Mock PA, et al. Sexual, reproductive and contraceptive risk factors for carcinoma-in-situ of the uterine cervix in Sydney. Med J Aust. 1989; 150:125-30.

15. Chaouki N, Bosch FX, Muñoz N, Meijer CJ, El Gueddari B, El Ghazi A, et al. The viral origin of cervical cancer in Rabat, Morocco. Int J Cancer. 1998; 75:546-54.

16. Chen MT, Cook LS, Daling JR, Weiss NS. Incomplete pregnancies and risk of ovarian cancer (Washington, United States). Cancer Causes Control. 1996; 7:415-20.

17. Chen Y, Wu PC, Lang JH, Ge WJ, Hartge P, Brinton LA. Risk factors for epithelial ovarian cancer in Beijing, China. Int J Epidemiol. 1992; 21:23-9.

18. Chiaffarino F, Pelucchi C, Parazzini F, Negri E, Franceschi S, Talamini R, et al. Reproductive and hormonal factors and ovarian cancer. Ann Oncol. 2001; 12:337-41.

19. Cusimano R, Dardanoni G, Dardanoni L, La Rosa M, Pavone G, Tumino R, et al. Risk factors of female cancers in Ragusa population (Sicily)--1. Endometrium and cervix uteri cancers. Eur J Epidemiol. 1989; 5:363-71.

20. Cuzick J, Sasieni P, Singer A. Risk factors for invasive cervix cancer in young women. Eur J Cancer. 1996; 32a:836-41.

21. Cuzick J, Singer A, De Stavola BL, Chomet J. Case-control study of risk factors for cervical intraepithelial neoplasia in young women. Eur J Cancer. 1990; 26:684-90.

22. Dossus L, Allen N, Kaaks R, Bakken K, Lund E, Tjonneland A, et al. Reproductive risk factors and endometrial cancer: the European Prospective Investigation into Cancer and Nutrition. Int J Cancer. 2010; 127:442-51.

23. Ebeling K, Nischan P, Schindler C. Use of oral contraceptives and risk of invasive cervical cancer in previously screened women. Int J Cancer. 1987; 39:427-30.

24. Elwood JM, Cole P, Rothman KJ, Kaplan SD. Epidemiology of endometrial cancer. Journal of the National Cancer Institute. 1977; 59:1055-60.

25. Franceschi S, Rajkumar T, Vaccarella S, Gajalakshmi V, Sharmila A, Snijders PJ, et al. Human papillomavirus and risk factors for cervical cancer in Chennai, India: a case-control study. Int J Cancer. 2003; 107:127-33.

26. Green J, Berrington de Gonzalez A, Sweetland S, Beral V, Chilvers C, Crossley B, et al. Risk factors for adenocarcinoma and squamous cell carcinoma of the cervix in women aged 20-44 years: the UK National Case-Control Study of Cervical Cancer. Br J Cancer. 2003; 89:2078-86.

27. Greggi S, Parazzini F, Paratore MP, Chatenoud L, Legge F, Mancuso S, et al. Risk factors for ovarian cancer in central Italy. Gynecol Oncol. 2000; 79:50-4.

28. Gwinn ML, Lee NC, Rhodes PH, Layde PM, Rubin GL. Pregnancy, breast feeding, and oral contraceptives and the risk of epithelial ovarian cancer. J Clin Epidemiol. 1990; 43:559-68.

29. Hammouda D, Muñoz N, Herrero R, Arslan A, Bouhadef A, Oublil M, et al. Cervical carcinoma in Algiers, Algeria: human papillomavirus and lifestyle risk factors. Int J Cancer. 2005; 113:483-9.

30. Harris R, Whittemore AS, Itnyre J. Characteristics relating to ovarian cancer risk: collaborative analysis of 12 US case-control studies. III. Epithelial tumors of low malignant potential in white women. Collaborative Ovarian Cancer Group. Am J Epidemiol. 1992; 136:1204-11.

31. Harris RW, Brinton LA, Cowdell RH, Skegg DC, Smith PG, Vessey MP, et al. Characteristics of women with dysplasia or carcinoma in situ of the cervix uteri. Br J Cancer. 1980; 42:359-69.

32. Hartge P, Schiffman MH, Hoover R, McGowan L, Lesher L, Norris HJ. A case-control study of epithelial ovarian cancer. Am J Obstet Gynecol. 1989; 161:10-6.

33. Hinkula M, Pukkala E, Kyyrönen P, Laukkanen P, Koskela P, Paavonen J, et al. A population-based study on the risk of cervical cancer and cervical intraepithelial neoplasia among grand multiparous women in Finland. Br J Cancer. 2004; 90:1025-9.

34. Horn-Ross PL, John EM, Canchola AJ, Stewart SL, Lee MM. Phytoestrogen intake and endometrial cancer risk. J Natl Cancer Inst. 2003; 95:1158-64.

35. John EM, Koo J, Horn-Ross PL. Lifetime physical activity and risk of endometrial cancer. Cancer Epidemiol Biomarkers Prev. 2010; 19:1276-83.

36. Jones CJ, Brinton LA, Hamman RF, Stolley PD, Lehman HF, Levine RS, et al. Risk factors for in situ cervical cancer: results from a case-control study. Cancer Res. 1990; 50:3657-62.

37. Jussawalla DJ, Deshpande VA, Standfast SJ. Assessment of risk patterns in cancer of the cervix: a comparison between greater Bombay and western countries. Int J Cancer. 1971; 7:259-68.

38. Jussawalla DJ, Yeole BB. Epidemiology of cancer of the cervix in greater Bombay. J Surg Oncol. 1984; 26:53-62.

39. La Vecchia C, Franceschi S, Gallus G, Decarli A, Colombo E, Mangioni C, et al. Oestrogens and obesity as risk factors for endometrial cancer in Italy. Int J Epidemiol. 1982; 11:120-6.

40. La Vecchia C, Franceschi S, Gallus G, Decarli A, Liberati A, Tognoni G. Incessant ovulation and ovarian cancer: a critical approach. Int J Epidemiol. 1983; 12:161-4.

41. Lambe M, Wuu J, Weiderpass E, Hsieh CC. Childbearing at older age and endometrial cancer risk (Sweden). Cancer Causes Control. 1999; 10:43-9.

42. Lesko SM, Rosenberg L, Kaufman DW, Stolley P, Warshauer ME, Lewis JL, Jr., et al. Endometrial cancer and age at last delivery: evidence for an association. Am J Epidemiol. 1991; 133:554-9.

43. McGowan L, Parent L, Lednar W, Norris HJ. The woman at risk for developing ovarian cancer. Gynecol Oncol. 1979; 7:325-44.

44. McPherson CP, Sellers TA, Potter JD, Bostick RM, Folsom AR. Reproductive factors and risk of endometrial cancer. The Iowa Women's Health Study. Am J Epidemiol. 1996; 143:1195-202.

45. Mogren I, Stenlund H, Högberg U. Long-term impact of reproductive factors on the risk of cervical, endometrial, ovarian and breast cancer. Acta Oncol. 2001; 40:849-54.

46. Moorman PG, Calingaert B, Palmieri RT, Iversen ES, Bentley RC, Halabi S, et al. Hormonal risk factors for ovarian cancer in premenopausal and postmenopausal women. Am J Epidemiol. 2008; 167:1059-69.

47. Nasca PC, Greenwald P, Chorost S, Richart R, Caputo T. An epidemiologic case-control study of ovarian cancer and reproductive factors. Am J Epidemiol. 1984; 119:705-13.

48. Neale RE, Darlington S, Murphy MF, Silcocks PB, Purdie DM, Talbäck M. The effects of twins, parity and age at first birth on cancer risk in Swedish women. Twin Res Hum Genet. 2005; 8:156-62.

49. Newton R, Ziegler J, Casabonne D, Beral V, Mbidde E, Carpenter L, et al. A case-control study of cancer of the uterine cervix in Uganda. Eur J Cancer Prev. 2007; 16:555-8.

50. Parazzini F, Chatenoud L, La Vecchia C, Negri E, Franceschi S, Bolis G. Determinants of risk of invasive cervical cancer in young women. Br J Cancer. 1998; 77:838-41.

51. Parazzini F, La Vecchia C, Negri E, Cecchetti G, Fedele L. Reproductive factors and the risk of invasive and intraepithelial cervical neoplasia. Br J Cancer. 1989; 59:805-9.

52. Parazzini F, Vecchia CL, Negri E, Fedele L, Franceschi S, Gallotta L. Risk factors for cervical intraepithelial neoplasia. Cancer. 1992; 69:2276-82.

53. Pfeiffer RM, Mitani A, Landgren O, Ekbom A, Kristinsson SY, Björkholm M, et al. Timing of births and endometrial cancer risk in Swedish women. Cancer Causes Control. 2009; 20:1441-9.

54. Pocobelli G, Doherty JA, Voigt LF, Beresford SA, Hill DA, Chen C, et al. Pregnancy history and risk of endometrial cancer. Epidemiology. 2011; 22:638-45.

55. Polychronopoulou A, Tzonou A, Hsieh CC, Kaprinis G, Rebelakos A, Toupadaki N, et al. Reproductive variables, tobacco, ethanol, coffee and somatometry as risk factors for ovarian cancer. Int J Cancer. 1993; 55:402-7.

56. Purdie D, Green A, Bain C, Siskind V, Ward B, Hacker N, et al. Reproductive and other factors and risk of epithelial ovarian cancer: an Australian case-control study. Survey of Women's Health Study Group. Int J Cancer. 1995; 62:678-84.

57. Riman T, Dickman PW, Nilsson S, Correia N, Nordlinder H, Magnusson CM, et al. Risk factors for invasive epithelial ovarian cancer: results from a Swedish case-control study. Am J Epidemiol. 2002; 156:363-73.

58. Salazar-Martinez E, Lazcano-Ponce EC, Gonzalez Lira-Lira G, Escudero-De los Rios P, Salmeron-Castro J, Hernandez-Avila M. Reproductive factors of ovarian and endometrial cancer risk in a high fertility population in Mexico. Cancer Res. 1999; 59:3658-62.

59. Shu XO, Brinton LA, Gao YT, Yuan JM. Population-based case-control study of ovarian cancer in Shanghai. Cancer Res. 1989; 49:3670-4.

60. Silins I, Wang X, Tadesse A, Jansen KU, Schiller JT, Avall-Lundqvist E, et al. A population-based study of cervical carcinoma and HPV infection in Latvia. Gynecol Oncol. 2004; 93:484-92.

61. Thakur A, Gupta B, Gupta A, Chauhan R. Risk factors for cancer cervix among rural women of a hilly state: a case-control study. Indian J Public Health. 2015; 59:45-8.

62. Titus-Ernstoff L, Perez K, Cramer DW, Harlow BL, Baron JA, Greenberg ER. Menstrual and reproductive factors in relation to ovarian cancer risk. Br J Cancer. 2001; 84:714-21.

63. Trabert B, Troisi R, Grotmol T, Ekbom A, Engeland A, Gissler M, et al. Associations of pregnancy-related factors and birth characteristics with risk of endometrial cancer: A Nordic population-based case-control study. Int J Cancer. 2020; 146:1523-31.

64. Ursin G, Pike MC, Preston-Martin S, d'Ablaing G, 3rd, Peters RK. Sexual, reproductive, and other risk factors for adenocarcinoma of the cervix: results from a population-based case-control study (California, United States). Cancer Causes Control. 1996; 7:391-401.

65. VanEenwyk J, Davis FG, Bowen PE. Dietary and serum carotenoids and cervical intraepithelial neoplasia. Int J Cancer. 1991; 48:34-8.

66. Wernli KJ, Ray RM, Gao DL, De Roos AJ, Checkoway H, Thomas DB. Menstrual and reproductive factors in relation to risk of endometrial cancer in Chinese women. Cancer Causes Control. 2006; 17:949-55.

67. Whiteman DC, Siskind V, Purdie DM, Green AC. Timing of pregnancy and the risk of epithelial ovarian cancer. Cancer Epidemiol Biomarkers Prev. 2003; 12:42-6.

68. Whittemore AS, Harris R, Itnyre J, Halpern J. Characteristics relating to ovarian cancer risk: collaborative analysis of 12 US case-control studies. I. Methods. Collaborative Ovarian Cancer Group. Am J Epidemiol. 1992; 136:1175-83.

69. Whittemore AS, Wu ML, Paffenbarger RS, Jr., Sarles DL, Kampert JB, Grosser S, et al. Personal and environmental characteristics related to epithelial ovarian cancer. II. Exposures to talcum powder, tobacco, alcohol, and coffee. Am J Epidemiol. 1988; 128:1228-40.

70. Wu AH, Pearce CL, Lee AW, Tseng C, Jotwani A, Patel P, et al. Timing of births and oral contraceptive use influences ovarian cancer risk. Int J Cancer. 2017; 141:2392-9.

71. Wynder EL, Escher GC, Mantel N. An epidemiological investigation of cancer of the endometrium. Cancer. 1966; 19:489-520.

72. Yang HP, Wentzensen N, Trabert B, Gierach GL, Felix AS, Gunter MJ, et al. Endometrial cancer risk factors by 2 main histologic subtypes: the NIH-AARP Diet and Health Study. Am J Epidemiol. 2013; 177:142-51.

73. Ylitalo N, Sørensen P, Josefsson A, Frisch M, Sparén P, Pontén J, et al. Smoking and oral contraceptives as risk factors for cervical carcinoma in situ. Int J Cancer. 1999; 81:357-65.

74. Yoo KY, Kang D, Koo HW, Park SK, Kim DH, Park NH, et al. Risk factors associated with uterine cervical cancer in Korea: a case-control study with special reference to sexual behavior. J Epidemiol. 1997; 7:117-23.

75. Zhang ZF, Parkin DM, Yu SZ, Estève J, Yang XZ. Risk factors for cancer of the cervix in a rural Chinese population. Int J Cancer. 1989; 43:762-7.

76. Zondervan KT, Carpenter LM, Painter R, Vessey MP. Oral contraceptives and cervical cancer--further findings from the Oxford Family Planning Association contraceptive study. Br J Cancer. 1996; 73:1291-7.
